# Therapeutic Effect of TSG-6 Engineered iPSC-Derived MSCs on Experimental Periodontitis in Rats: A Pilot Study

**DOI:** 10.1371/journal.pone.0100285

**Published:** 2014-06-30

**Authors:** Heng Yang, Raydolfo M. Aprecio, Xiaodong Zhou, Qi Wang, Wu Zhang, Yi Ding, Yiming Li

**Affiliations:** 1 State Key Laboratory of Oral Diseases, Sichuan University, Chengdu, Sichuan, China; 2 Center for Dental Research, Loma Linda University School of Dentistry, Loma Linda, California, United States of America; 3 Department of Periodontology, West China Hospital of Stomatology, Sichuan University, Chengdu, Sichuan, China; Boston University, United States of America

## Abstract

**Background:**

We derived mesenchymal stem cells (MSCs) from rat induced pluripotent stem cells (iPSCs) and transduced them with tumor necrosis factor alpha-stimulated gene-6 (TSG-6), to test whether TSG-6 overexpression would boost the therapeutic effects of iPSC-derived MSCs in experimental periodontitis.

**Methods:**

A total of 30 female Sprague-Dawley (SD) rats were randomly divided into four groups: healthy control group (Group-N, n = 5), untreated periodontitis group (Group-P, n = 5), iPS-MSCs-treated and iPSC-MSCs/TSG-6-treated periodontitis groups (Group-P1 and P2, n = 10 per group). Experimental periodontitis was established by ligature and infection with *Porphyromonas gingivalis* around the maxillae first molar bilaterally. MSC-like cells were generated from rat iPSCs, and transducted with TSG-6. iPSC-MSCs or iPSC-MSCs/TSG-6 were administrated to rats in Group-P1 or P2 intravenously and topically, once a week for three weeks. Blood samples were obtained one week post-injection for the analysis of serum pro-inflammatory cytokines. All animals were killed 3 months post-treatment; maxillae were then dissected for histological analysis, tartrate-resistant acid phosphatase (TRAP) staining, and morphological analysis of alveolar bone loss.

**Results:**

Administration of iPSC-MSC/TSG-6 significantly decreased serum levels of IL-1β and TNF-α in the Group-P2 rats (65.78 pg/ml and 0.56 pg/ml) compared with those in Group-P (168.31 pg/ml and 1.15 pg/ml respectively) (p<0.05). Both alveolar bone loss and the number of TRAP-positive osteoclasts showed a significant decrease in rats that received iPSC-MSC/TSG-6 treatment compared to untreated rats in Group-P (p<0.05),

**Conclusions:**

We demonstrated that overexpression of TSG-6 in rat iPSC-derived MSCs were capable of decreasing inflammation in experimental periodontitis and inhibiting alveolar bone resorption. This may potentially serve as an alternative stem-cell-based approach in the treatment and regeneration of periodontal tissues.

## Introduction

Periodontitis is a chronic infectious disease, leading to periodontal tissue inflammation, attachment loss, alveolar bone resorption, and eventually tooth loss [1]. To date, several therapies, such as mechanical and chemical root conditioning, implantation of autografts, allografts and alloplastic materials, growth factors, guided tissue regeneration, and various combinations of these approaches, have been used in clinical practice with the aim of achieving true periodontal regeneration [2]–[5]. However, the clinical results vary widely and are often unpredictable. Currently, stem cell biology has become an important field for the understanding of regenerative medicine.

Multipotent mesenchymal stem cells (MSCs) are a population of postnatal stem cells that have been successfully isolated from various human tissues. MSCs have been shown to possess a self-renewing capacity and can differentiate several cell lineages, including osteocytes, chondrocytes, and adipocytes [6]–[9]. MSCs are thus considered an attractive candidate for regenerative therapy. Recent reports indicate that MSCs are important guardian cells for modulating inflammation. Certain therapeutic effects of the cells seen in animal models are believed to be the result of MSCs being activated by signals from injured tissues to secrete anti-inflammatory factors [10], [11]. Among these factors, the most interesting was tumor necrosis factor alpha-stimulated gene-6 (TSG-6) [12], which has been extensively studied in articular joint diseases [13]–[15]. The anti-inflammatory and chondroprotective effects of TSG-6 have been reported in numerous animal models [16]–[19]. However, the combined effect of MSCs and TSG-6 has not been investigated.

Although MSCs are recognized as having a great potential for regeneration, extended *in vitro* culture reduces the differentiation potential of MSCs, which limits their therapeutic efficacy [20]. Successful generation of induced pluripotent stem cells (iPSCs) by Yamanaka and co-workers [21], which can be expanded to large numbers before *in vitro* differentiation and transplantation, is an option to overcome such limitations seen with MSCs.

The objective of the present study was to derive MSCs from rat iPSCs and transduce them with TSG-6 to test our hypothesis that TSG-6 overexpression will boost the anti-inflammatory effects of iPSC-derived MSCs in experimental periodontitis.

## Materials and Methods

### iPSC-derived MSC (iPSC-MSC) generation

Based on our previous study, rat iPSCs were reprogrammed from female rat embryonic fibroblasts by transducing them with Oct4, Sox2, Myc, and Klf4-expressing lentiviral vectors [22]. iPSCs in passage 5 were cultured in a gelatin-coated six-well plate with Minimum Essential Medium (MEM; Gibco, Life Technologies, Grand Island, NY, USA) supplemented with 2% fetal bovine serum (FBS; Fisher Scientific, Pittsburgh, PA, USA), 1% Penicillin/Stremycin (Gibco), 5% knockout serum replacement (Gibco), 1% platelet-derived growth factor, 1% fibroblast growth factor-2, and 0.1% epidermal growth factor. Cytokines were purchased from ProSpec (East Brunswick, NJ, USA). Cells were cultured under hypoxic conditions [23] by placing culture plates in a Hypoxia Chamber (Stem cell Technologies, Vancouver, British Columbia, Canada) that was flushed with mixed air composed of 92% N_2_, 3% O_2_, and 5% CO_2_. Media were replaced every another day. Cells were cultured and passaged until homogeneous fibroblastic morphology appeared.

### Flow cytometry assay

The representative markers characteristic of rat MSCs were confirmed with flow cytometry following the previous protocol [22]. Briefly, cells were harvested by Accutase treatment (Innovative Cell Technologies, San Diego, CA, USA) and fixed for 30 minutes at room temperature in fixation buffer and permeabilization buffer (eBiosciences, San Diego, CA, USA). After washing, cells were stained at room temperature for 2 hours with antibodies followed by washing twice with permeabilization buffer. The following conjugated antibodies were used: fluorescein isothiocyanate (FITC)-conjugated against CD45, and PE-conjugated against CD29 and CD90 (BD Biosciences, San Jose, CA, USA). Flow cytometric analysis was performed using FACS Aria II (BD Biosciences) with a 488 nm laser. For each sample 30,000 events were collected.

### Transfection and overexpression TSG-6 in iPSC-derived MSCs *in vitro*

The construction of the lentiviral vector was performed according to our previous study [24]. Subcultured iPSC-MSCs were transfected with TSG-6 lentiviral vector for 24 hours. Total RNA was isolated using RNeasy Mini Kit (QIAGEN, Valencia, CA, USA). First-strand complimentary DNA (cDNA) was synthesized using Super-Script III First-Strand Synthesis System and the OligodT primer (Invitrogen, Life Technologies, Grand Island, NY, USA). Real-time amplification was performed using Taqman Universal PCR master mix (Applied Biosystems, Grand Island, NY, USA) on RT-PCR System (ViiA 7 real-time PCR system, Applied Biosystems). An 18s ribosomal RNA (rRNA) probe (Taqman Gene Expression Assays ID, Hs03003631_g1) was used for normalization of gene expression. The PCR products were resolved by electrophoresis on 1.5% (w/v) agarose gels containing ethidium bromide (0.5 mg/mL) to visualize the PCR products.

### Animal model of periodontitis and treatment

The experimental protocols were approved by Loma Linda University Institutional Animal Care and Use Committee (IACUC, permit number: 699310-2938). A total of 30, 7-week-old female Sprague-Dawley (SD) rats (SAS-SD rat; Charles River Laboratories International, Inc., Wilmington, MA, USA) were used. Animals were randomly assigned to four groups: healthy control (Group-N, n = 5), untreated periodontitis (Group-P, n = 5), iPSC-MSCs-treated periodontitis (Group-P1, n = 10), or iPSC-MSCs/TSG-6-treated periodontitis (Group-P2, n = 10).

Following anesthesia by intraperitoneal (i.p.) injection of ketamine/xylazine (Clipper Distributing Company, St. Joseph, MO, USA and Akorn, Inc., Decatur, IL, USA), orthodontic wire of 0.2 mm in diameter was bilaterally ligatured around the first molar of the rat maxilla in Group P, P1, and P2. Cultures of *Porphyromonas gingivalis* (*P. gingivalis* 381, ATCC, Manassas, VA, USA) at 10^10^ cfu/mL were inoculated into the oral cavity four times a week for 4 weeks to establish experimental periodontitis. The wires were then removed, and the treatment was applied as follows: rats in Groups P1 and P2 received iPSC-MSCs and iPSC-MSCs/TSG-6, respectively, through both systemic and topical injections. For systemic injections, rats received 5×10^6^ cells in 200 µL of culture media via the tail vein; for topical injections, 10^6^cells in 20 µL of matrigel (BD Biosciences) were delivered around the maxilla first molar. Cells were injected once a week for 3 weeks. All animals were killed 3 months post-injection.

### ELISA analysis

Rat tail blood samples were collected one week post-cell injections and centrifuged at 1,000 rpm for 15 minutes. The supernatant was collected and stored at −20°C. Samples were thawed at room temperature before analysis. Serum concentrations of interleukin-1β (IL-1β) and tumor necrosis factor-α (TNF-α) were analyzed using enzyme-linked immunosorbent assay (ELISA) (Rat quantikine ELISA kit, R&D, Minneapolis, MN, USA and BlueGene ELISA kit, Life Sciences Advanced Technologies Inc, Saint Petersburg, FL, USA).

### TRAP staining

The rats were killed and the maxillae were dissected and fixed in 10% formalin for 48 hours. The left maxillae were decalcified in 15% EDTA solution for 2 weeks. Specimens were paraffin-embedded; and mesial-distal orientated 5 µm sections were prepared. Alternate slides were stained with hematoxylin and eosin (H&E) for descriptive histology. For enumeration of osteoclasts, tartrate-resistant acid phosphatase (TRAP) staining was carried out using a leukocyte acid phosphatase kit (Sigma, St. Louis, MO, USA). Active osteoclasts were defined as multinucleated (≥3) TRAP-positive cells in contact with the bone surface located between the first and second molars. Four continual sections were used for the enumeration of osteoclasts. Images were captured with an Aperio Scan Scope (Aperio Technologies, Vista, CA, USA), and cells were counted using Image J software (NIHImage).

### Morphometric evaluation of alveolar bone loss

The 10% formalin-fixed right maxillae were dehydrated by passing through a graded series of ethanol solutions (from 30% to 100%). Specimens were incubated in a desiccator for 48 hours then coated with platinum and examined under a scanning electron microscope (VEGA II Tescan, Cranberry TWP., PA, USA) at an accelerating voltage of 10 kV. Alveolar bone loss was evaluated morphometrically by measuring the distance between the cemento-enamel junction (CEJ) and the palatal alveolar bone crest (ABC) at nine sites [25] from first to second molar for three times. For each animal, alveolar bone loss was defined as the mean of nine measurements. Images were analyzed using Image J software.

### Statistical analysis

Data were expressed as mean±standard error of the mean (SEM). Comparison of parameters among the groups was performed using one-way analysis of variance (ANOVA) using SPSS software (SPSS 17.0, Chicago, IL, USA). A p-value of <0.05 was considered statistically significant.

## Results

### Derivation of MSCs from rat iPSCs

**Figure 1:**
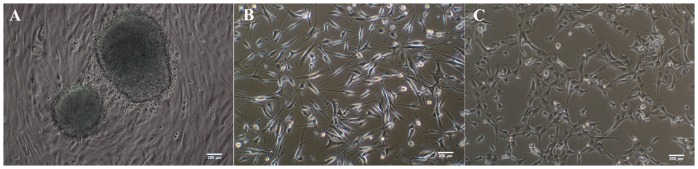
Cell culture: Morphology of cells under light microscopy. (A) Rat induced pluripotent stem cells (iPSCs). (B) Rat bone marrow mesenchymal stem cells (BM-MSCs), passage 3. (C) iPSC derived-MSCs, passage 3. Scale bar, 200 µm.

**Figure 2:**
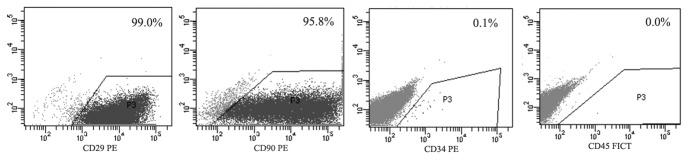
Flow cytometry assay. The characteristic cell surface makers of rat MSCs were detected by FACS. The iPSC derived MSCs at passage 5 revealed positivity for CD29 and CD90, negativity for CD34 and CD45.

After five passages by culturing in MSC culture medium, rat iPSCs began to form MSC-like cells, which were similar to the morphology of rat BM-MSCs (Fig. 1). Subsequently, flow cytometry analysis showed that these cells expressed CD29 and CD90, but were negative for CD34 and CD45 (Fig. 2).

### Overexpression of TSG-6 in iPSC-MSCs *in vitro*

**Figure 3:**
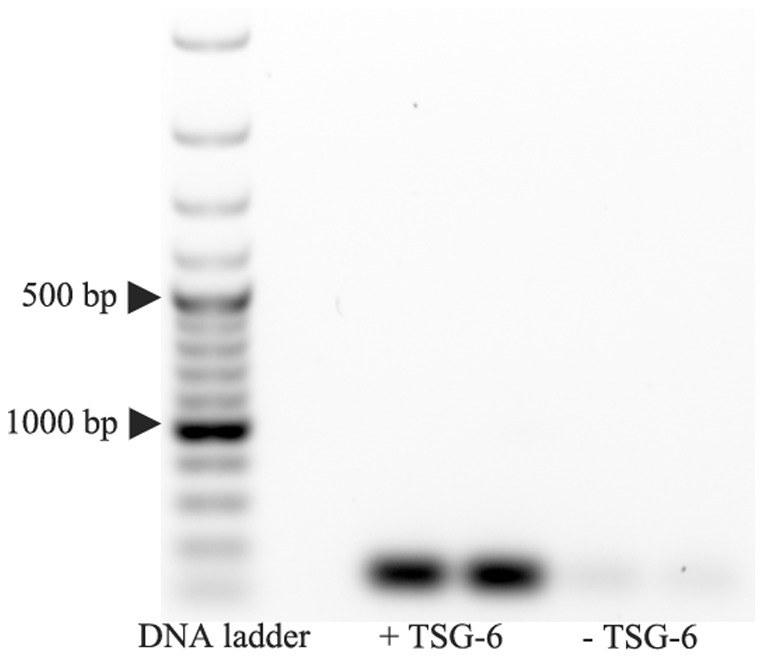
Overexpression TSG-6 in iPSC-derive MSCs in vitro. Overexpression TSG-6 in iPSC-MSCs in vitro. After 24 hrs transfection, TSG-6 was overexpressed in iPSC-MSCs in vitro, detected by PCR.

After lentiviral transfection for 24 hours, iPSC-MSCs were harvested using Accutasefor total mRNA extraction and cDNA synthesis for RT-PCR. As a result, TSG-6 expression increased approximately 18-fold in transfected iPSC-MSCs, whereas TSG-6 expression in untransfected iPSC-MSCs was too low to be detected (Fig. 3).

### Suppression of inflammation by systemic and topical injections of iPSC-MSCs/TSG-6

**Figure 4:**
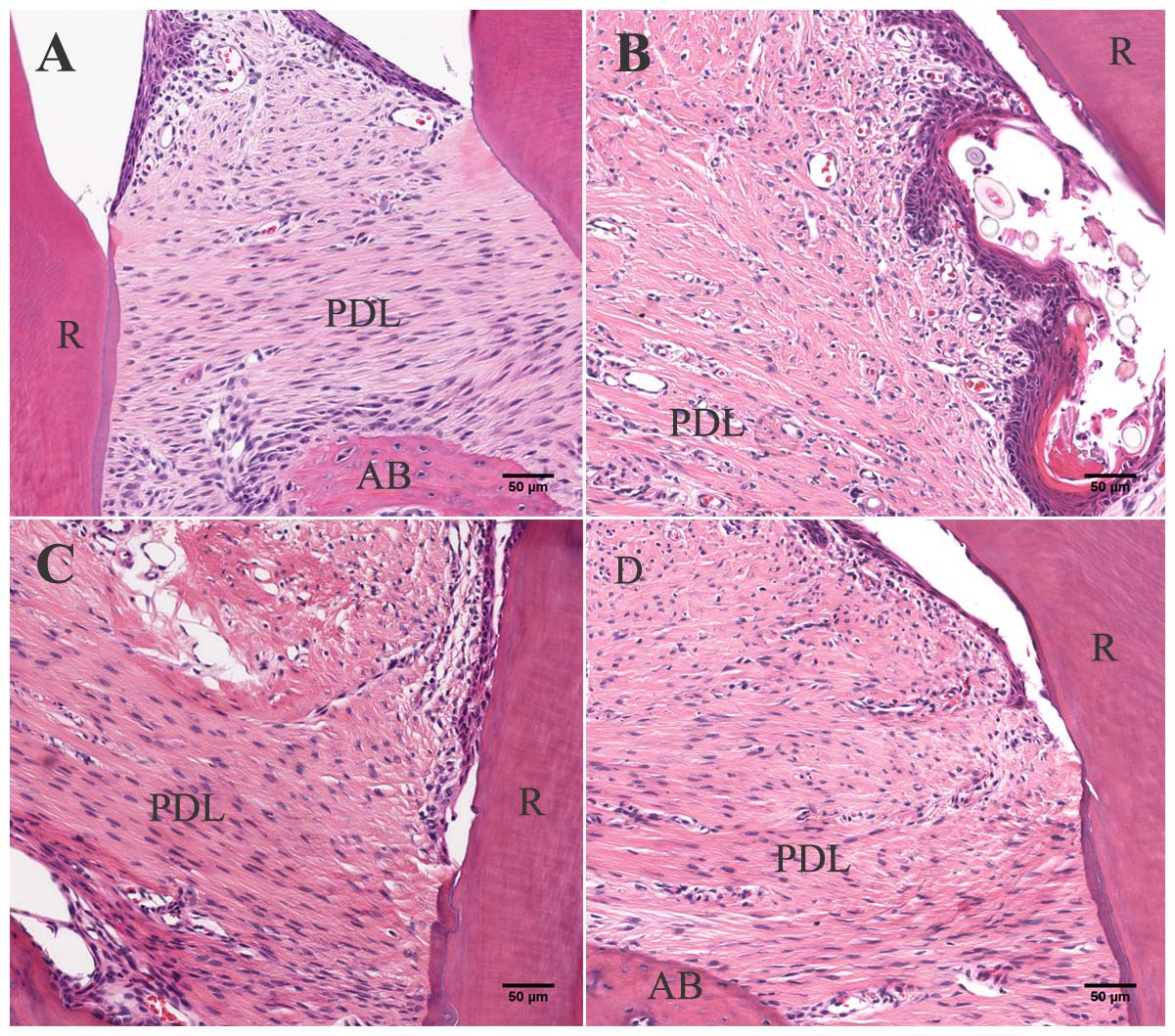
Histological analysis. Histological analysis showed severe inflammation in untreated periodontitis rats (B), while no inflammation was observed in healthy control animals (A); inflammatory infiltration in periodontal tissue decreased in rats received iPSC-MSCs (C) or iPSC-MSCs/TSG-6 (D) (H&E staining, scale bar, 50 µm; AB = alveolar bone, PDL = periodontal ligament, R = tooth root).

**Figure 5:**
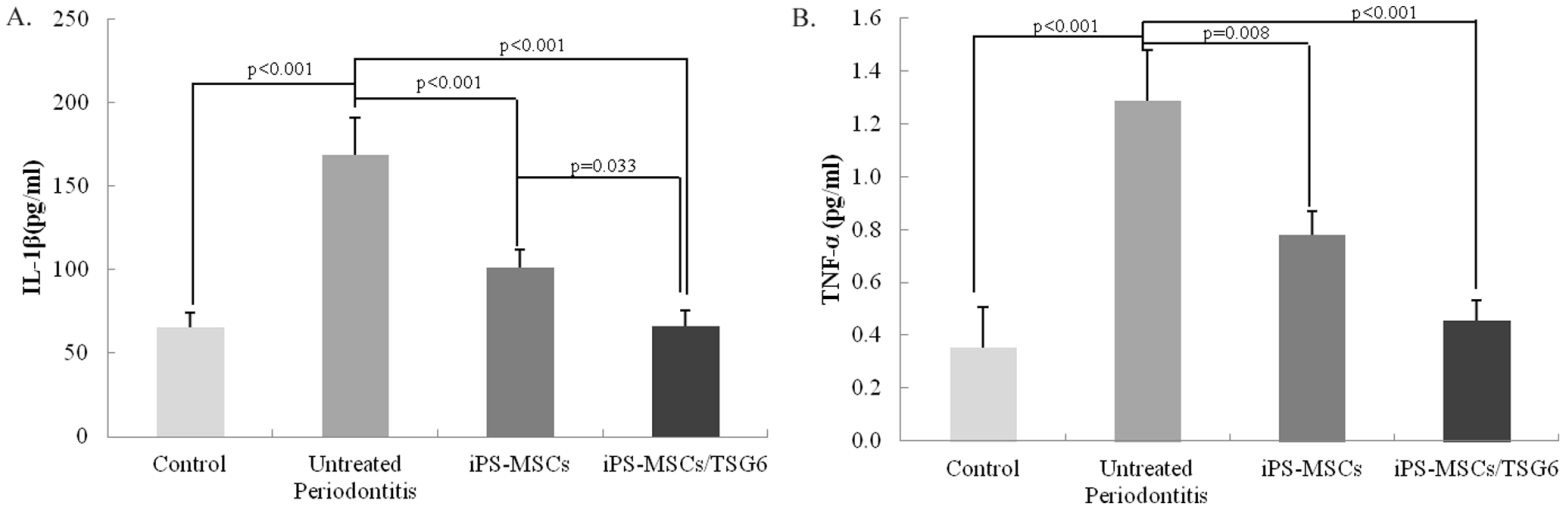
Pro-inflammation cytokine. Pro-inflammatory cytokine IL-1β and TNF-α in serum was detected by ELISA. The serum concentration of IL-1β and TNF-α showed a significant decrease after iPS-MSCs/TSG-6 treated when compared to untreated periodontitis group; and no significant differences compared to healthy control group.

The systemic administration of iPSC-MSCs and iPSC-MSCs/TSG-6 significantly reduced periodontal inflammation. Histologically, the infiltration of inflammatory cells in the periodontal tissues was markedly decreased in the iPSC-MSCs/TSG-6-treated group (Fig. 4). The production of proinflammatory cytokines was also significantly decreased in serum samples that were measured by ELISA (Fig. 5). IL-1β and TNF-α were significantly decreased in the iPSC-MSCs/TSG-6-treated group compared to the untreated periodontitis group (p<0.001); but no significant difference was detected compared to the healthy control group.

### Overexpression of TSG-6 inhibited osteoclast formation and alveolar bone loss

**Figure 6:**
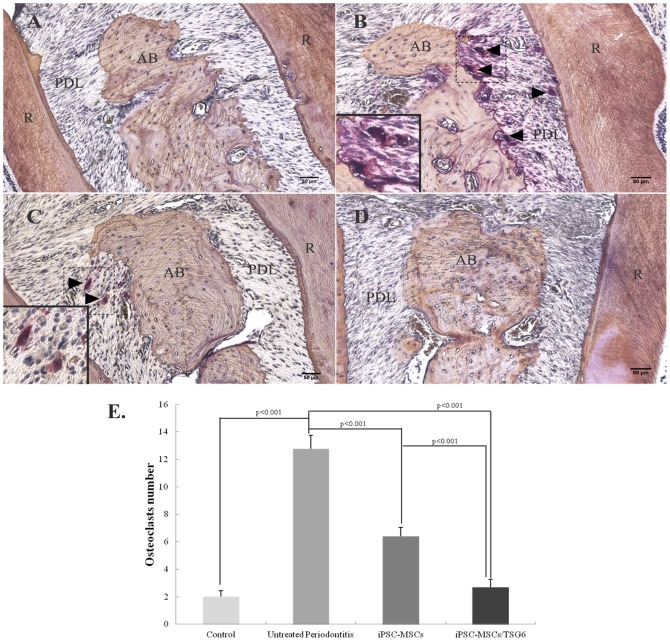
TRAP staining: Osteoclasts formation in different groups. (A) Healthy control group. (B) Untreated periodontitis group. (C) iPSC-MSCs treated periodontitis group. (D) iPSC-MSCs/TSG-6 treated periodontitis group. TRAP-positive osteoclasts in rat maxillae showed a significant decrease in iPSC-MSCs/TSG-6 treated group. (AB = alveolar bone, PDL = periodontal ligament, R = tooth root, black arrow = TRAP-positive osteoclasts) (TRAP staining, scale bar, 50 µm). (E) Quantitative analysis of TRAP-positive osteoclasts (nuclei≥3) showed a significantly decreased number of osteoclasts in iPSC-MSCs and iPSC-MSCs/TSG-6 treated group, compared to untreated periodontitis group; the decrease number of osteoclasts showed a significant differences between iPSC-MSCs/TSG-6 and iPSC-MSCs treated group.

**Figure 7:**
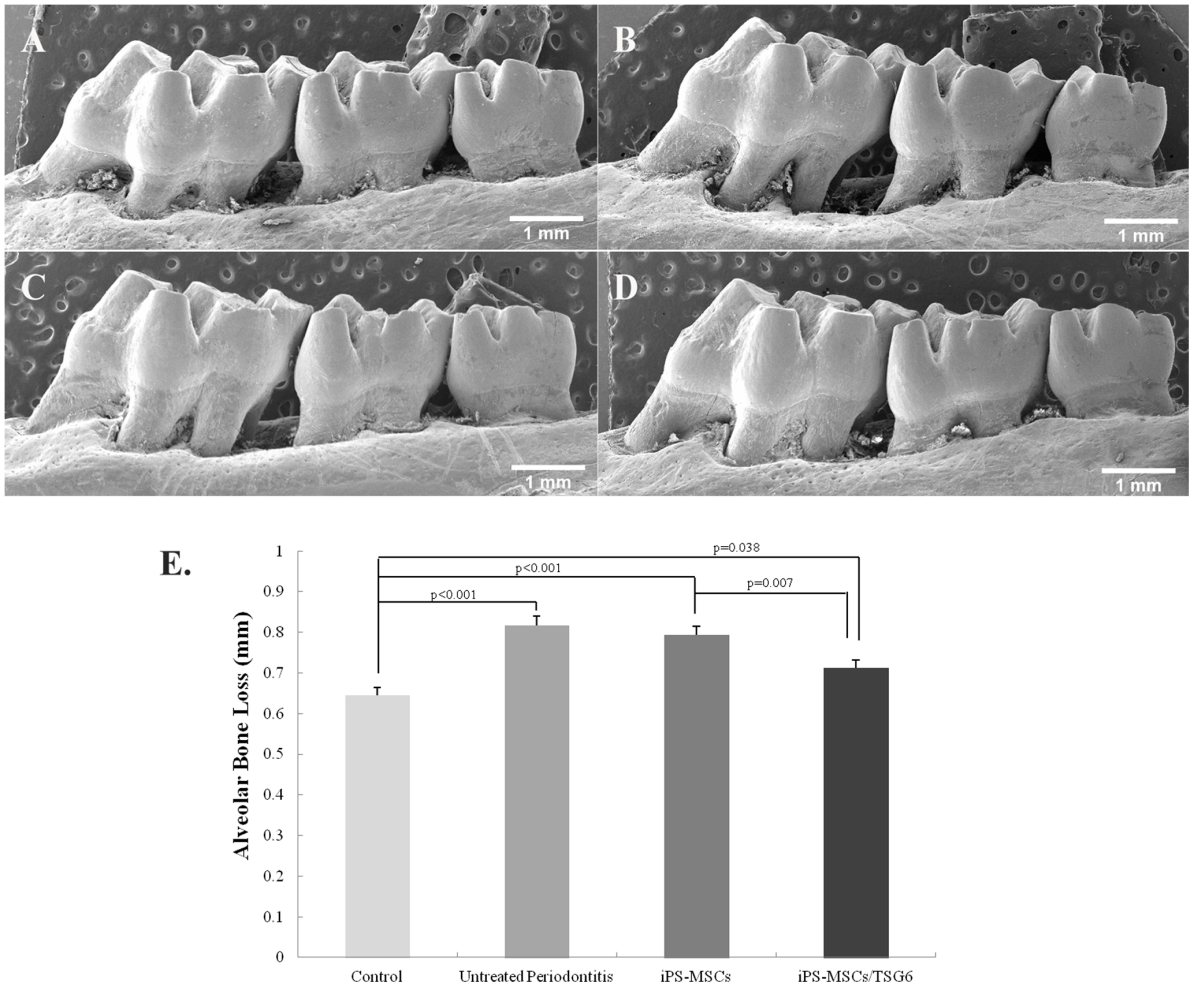
Alveolar bone loss: Alveolar bone loss analysis. (A) Healthy control group. (B) Untreated periodontitis group. (C) iPSC-MSCs treated periodontitis group. (D) iPSC-MSCs/TSG-6 treated periodontitis group. Scale bar, 1 mm. (E) Alveolar bone loss (ABL) was analyzed by measuring the distance between the cementum-enamel junction (CEJ) and the palatal alveolar bone crest (ABC) at 9 sites from first to second molar. The ABL in iPSC-MSCs/TSG-6 treated group showed a significant difference compared to the other groups.

TRAP-positive osteoclasts (nuclei≥3) in the iPSC-MSCs/TSG-6-treated group significantly decreased compared to the untreated periodontitis group three months post-treatment (Fig. 6). Alveolar bone analysis showed that rats from the untreated periodontitis group had significant bone loss compared to the healthy control group (p<0.001). Rats from the iPSC-MSCs/TSG-6 group had less bone loss than the untreated periodontitis group (p = 0.001) (Fig. 7).

## Discussion

In 2006, iPS cells were first generated from mouse embryonic fibroblasts and adult mouse tail-tip fibroblasts by transfecting four transcription factors: Oct3/4, Sox2, Klf4, and c-Myc [21]. iPS cells have comparable properties to embryonic stem cells in terms of morphology, proliferation, and gene expression *in vitro* and teratoma formation *in vivo*, without many of the ethical concerns associated with embryonic stem cells. iPS cells can be used to regenerate different tissues and differentiate to different cell lineages, which promises great potential for cell therapy and regenerative medicine [26]–[28]. iPS cells, derived from somatic cells and reprogrammed to the pluripotent state by the induced expression of defined transcription factors, can be expanded to large numbers before *in vitro* differentiation and transplantation. Consequently, deriving MSCs from iPSCs represents an important alternative to overcome the limitations seen with MSCs. Recent studies have reported functional MSCs derived from human iPSCs, which expressed characteristic MSC makers and differentiated into osteoblasts, adipocytes and chondrocytes, promoted vascular and muscle regeneration [29], or formed bones in mice after transplantation *in vivo* [30].

In the present study, in order to use iPSC-derived MSCs for treating experimental periodontitis, we attempted to generate rat iPSCs first. We previously reported successful reprograming of human and mouse cells to iPSCs by transducing with Oct4, Sox2, Myc, and Klf4-expressing lentiviral vectors [24]; in the present study the same strategy was used to generate iPSCs from rat embryonic fibroblasts. After culturing rat iPSCs in MSC media for five passages, MSC-like cells were derived from rat iPSCs, as shown by the expression of typical rat MSC surface makers (Figs. 1 to 3) [31]. Subcultured cells of iPSC-derived MSCs were successfully differentiated into osteoblasts, adipocytes, and chondrocytes *in vitro*.

TSG-6 is a 35 kDa inflammation-induced protein that was first discovered by differential screening of a cDNA library prepared from TNF-stimulated human FS-4 fibroblasts. It is not constitutively expressed in normal tissues or cells, but up-regulated in response to pro-inflammatory mediators, such as TNF, IL-1, and IL-6 [13], [14]. TSG-6 has been reported to have an anti-inflammatory effect in several animal models including arthritis, myocardial infarction, and chemical injury to cornea [15]–[17]; this finding has been attributed to its inhibitory effects on neutrophil migration and plasmin activity [32]–[34].

In the present study, TSG-6 was transfected into iPS-MSCs to examine the hypothesis that overexpression of TSG-6 would boost the anti-inflammatory effects of iPS-MSCs. Our data demonstrate that systemic and topical administration of TSG-6 engineered iPSC-MSCs significantly decreased the serum concentrations of proinflammatory cytokines, which indicates an anti-inflammatory effect in experimental periodontitis. In addition, histologic results showed less inflammatory infiltration in periodontal tissues after treatment. iPSC-MSCs without TSG-6 also had an anti-inflammatory effect on the experimental periodontitis which, however, was significantly weaker compared to the TSG-6-modified iPSC-MSC-treated group, indicating that TSG-6 enhanced the anti-inflammatory function of iPS-MSCs. The mechanism of the anti-inflammatory action of MSCs through secretion of TSG-6 has been indicated in Choi's study: MSCs introduced a negative feedback loop into the inflammatory response in which the MSCs and TSG-6 suppressed the initial production of pro-inflammatory cytokines (TNF-α and IL-1) from zymosan-activated macrophages [35]. Morphological analysis of alveolar bone loss (Fig. 7) also showed anti-bone resorption of TSG-6-modified iPS-MSCs by suppressing osteoclast formation and inhibiting alveolar bone resorption in this investigation. Previous study has shown that TSG-6 could regulate bone remodeling though the inhibition of osteoclast activity and the synergistic formation with osteoprotegerin [18], [19]. They indicated an autocrine mechanism of TSG-6 inhibiting osteoclasts activation; that is, osteoclast precursors and mature osteoclasts produced TSG-6 in response to pro-inflammatory cytokines (i.e. TNF-α, IL-1, and IL-6) and thereby limit their own ability to anchor to and resorb bone.

Previous work showed that the four factor-derived (Oct3/4, Sox2, Klf4, and c-Myc) iPSCs can cause tumor formation on reactivation of c-Myc [36]. My et al. [37] showed that rat iPSCs derived without c-Myc did not develop tumors, strongly suggesting that the presence of the *c-Myc* gene is a serious problem for their biomedical and clinical application. Thus c-Myc-free or non-viral reprogrammed iPSCs may be important for the future application in our further study. Although MSCs or TSG-6 have an anti-inflammatory effect, the combination of TSG-6 and iPSC-MSCs could enhance the therapeutic effect of MSCs, at the same time, iPSC-derived MSCs will maintain their multipotentiality to reconstruct periodontium destruction. Base on our pilot investigation, further studies on regulating periodontal regeneration using tissue-engineering approaches on bone defection models are in progress.

In summary, we demonstrated that overexpression of TSG-6 in rat iPSC-derived MSCs are capable of decreasing inflammation in experimental periodontitis, and inhibiting alveolar bone resorption, and may potentially serve as an alternative stem-cell-based approach in the treatment and regeneration of periodontal tissues.
